# Idiopathic Portal Vein Thrombosis in a Non-cirrhotic Patient: A Discussion of Management and a Review of Literature

**DOI:** 10.7759/cureus.17445

**Published:** 2021-08-25

**Authors:** Justin Y Ng, Sacha Reason, Jessica Y Ng

**Affiliations:** 1 Surgery, Gold Coast University Hospital, Southport, AUS; 2 Orthopaedics, The Bone Doctor.net, Mackay, AUS

**Keywords:** portal vein thrombosis, doacs and portal vein thrombosis, hepatobiliary surgeries, gastroentero-hepatology, therapeutic anticoagulation, gi radiology

## Abstract

Portal vein thrombosis (PVT) is most commonly seen in people with predisposing conditions such as cirrhosis, hepatobiliary malignancies, infectious or inflammatory abdominal disease, or haematologic disorders. However, the incidence of idiopathic portal vein thrombosis in non-cirrhotic people is low and approximately 25% of existing cases have no identifiable cause. If untreated, complications can include portal hypertension, a cavernous transformation of the portal vein, varices, septic thrombosis, or intestinal ischemia. We report the case of a 27-year-old female who presented to her general practitioner with two weeks of epigastric pain. She was referred for an upper abdominal USG and CT imaging, which identified portal vein thrombosis with a normal appearance of the gallbladder, liver, and spleen. Thrombophilia screen was negative for Factor V Leiden and prothrombin mutations and lupus anticoagulant. The tumour markers alpha-fetoprotein and carcinoembryonic antigen were also within normal limits. The patient was started on rivaroxaban indefinitely following advice from a vascular surgeon and haematologist. Subsequent follow-up imaging also revealed cavernous transformation of the portal vein. We present this case to discuss the diagnosis, management and treatment of this patient and to review the current evidence available in managing idiopathic portal vein thrombosis in non-cirrhotic patients, especially the role of anticoagulation in chronic cases.

## Introduction

The portal vein is a continuation of the superior mesenteric vein draining into the liver and is given its name upon its convergence with the splenic vein. Thrombosis can be partial or complete and most commonly occur secondary to primary or metastatic malignancies to the liver, cirrhosis from malignant and non-malignant diseases, inflammatory disorders such as Behçet syndrome, abdominal infections, and haematological disorders including myeloproliferative or coagulation disorders. The overall lifetime risk in the general population of developing portal vein thrombosis (PVT) is estimated to be approximately 1% [1] according to a previous study. Clinical manifestations of this condition vary. Symptoms can be vague and can include abdominal pain or fevers and patients can even be asymptomatic with the thrombosis found incidentally [2]. However, since PVT is commonly secondary to chronic liver diseases, patients often present with complications, such as variceal bleeding from portal hypertension or spontaneous bacterial peritonitis. The emphasis, in this case, is placed on the idiopathic nature of the diagnosis and the unique approach to management for this patient.

## Case presentation

A 27-year-old female presented to her general practitioner with two weeks of epigastric pain and new onset reflux. She had a notable medical history of an episode of unprovoked left leg deep venous thrombosis confirmed on USG three years prior. She was anticoagulated for six months then with rivaroxaban for an initial episode of unprovoked deep venous thrombosis. Investigations completed at the time included Protein C levels, which measured 1.46 units/mL (normal range 0.70 - 1.30 units/mL), and a Protein S assay, which measured 63% (normal range 50-135%), and coagulation studies, which were only significant for a thrombin clotting time of 12 seconds (normal range 13-21 seconds). The investigations did not reveal any causes for the episode of thrombosis and no further investigations were pursued at the time. Family history was only significant for her father who had an episode of provoked deep vein thrombosis due to trauma. The patient is a non-smoker and did not consume alcohol. She had no other prothrombotic risk factors such as recent operations, previous or current malignancies, recent long-haul travel or immobility, and was not currently or recently pregnant.

**Figure 1:**
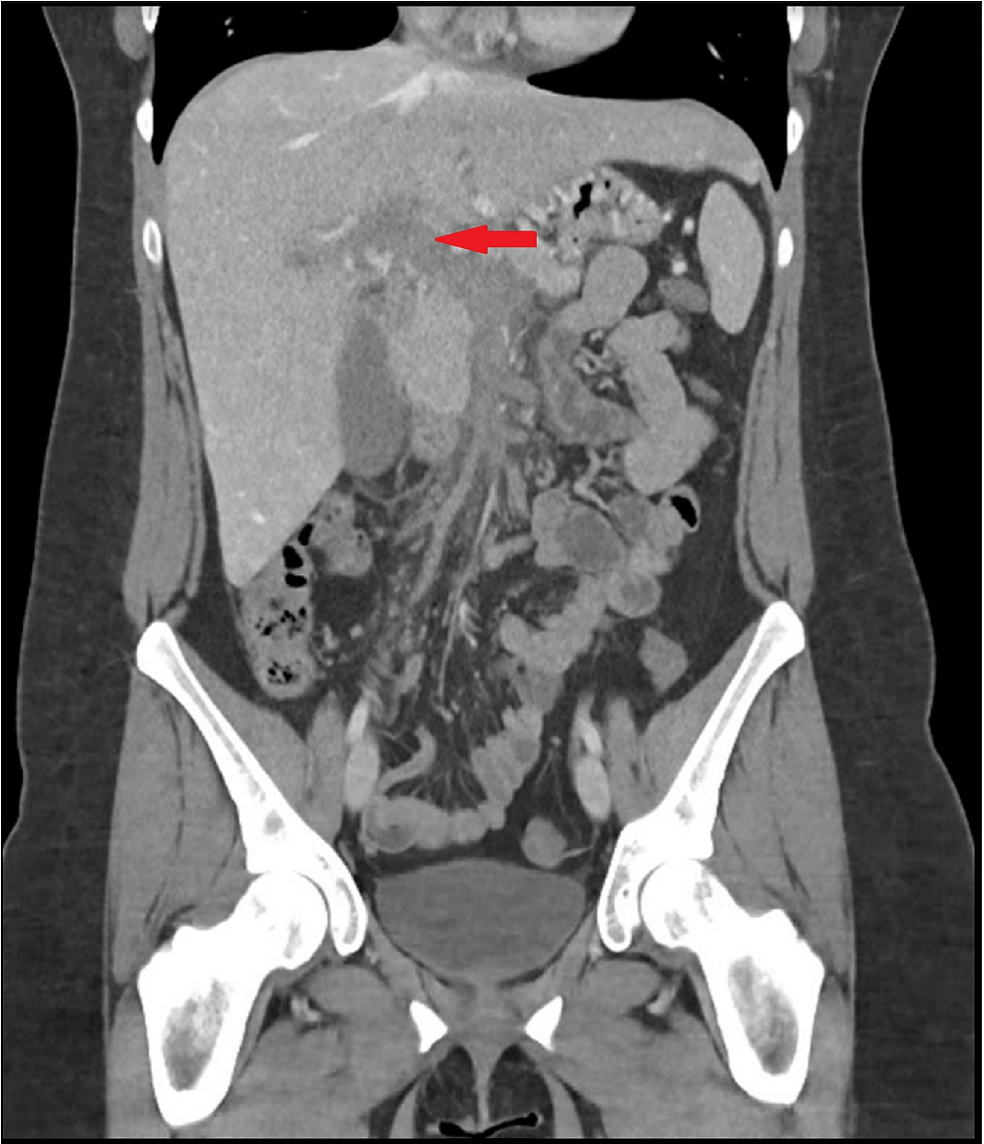
Initial CT imaging Coronal section of initial CT on diagnosis showing portal vein thrombosis

A USG and CT imaging of the abdomen demonstrated portal vein thrombosis with mesenteric congestion and small splenic hilar varices (Figure 1). The patient was prescribed rivaroxaban with an initial loading dosage of 15mg twice daily for three weeks, followed by 20mg once daily as per recommended guidelines. She reported partial improvement with her symptoms over the following weeks. 

Blood tests done during the initial investigation of the thrombosis were unremarkable. These blood tests included haemoglobin, which was 134 g/L, platelets of 194x10^9/L, total bilirubin of 8 umol/L, alkaline phosphatase of 85 units/L, gamma-glutamyltransferase measuring 28 units/L, alanine transaminase measuring 40 units/L, aspartate transaminase measuring 21 units/L, and a lipase level of 24 units/L. In addition to the normal liver function tests, she had no stigmata of chronic liver disease.

A thrombophilia screen was completed. Factor V Leiden G1691A mutation and Prothrombin G20210A mutation were not detected. Protein C (functional) was normal at 1.41 units/mL (normal range 0.70-2.00 units/mL) and Protein S (free antigen) was normal at 0.89 units/mL (0.55-2.00 units/mL). Lupus anticoagulant was not detected. Coagulation studies including prothrombin time, activated partial thromboplastin time, fibrinogen, and platelets were also within normal limits. Thrombin clotting time again was 12 seconds (normal range 13-21 seconds). These values did not explain the cause of recurrent thrombosis.

The patient was referred to a clinical haematologist for further investigation and advice on anticoagulation. Additional blood tests were ordered looking for rare causes of thrombosis. JAK2 mutations, paroxysmal nocturnal haematuria screen, and antithrombin III screens ran by the haematologist were normal. 

**Figure 2:**
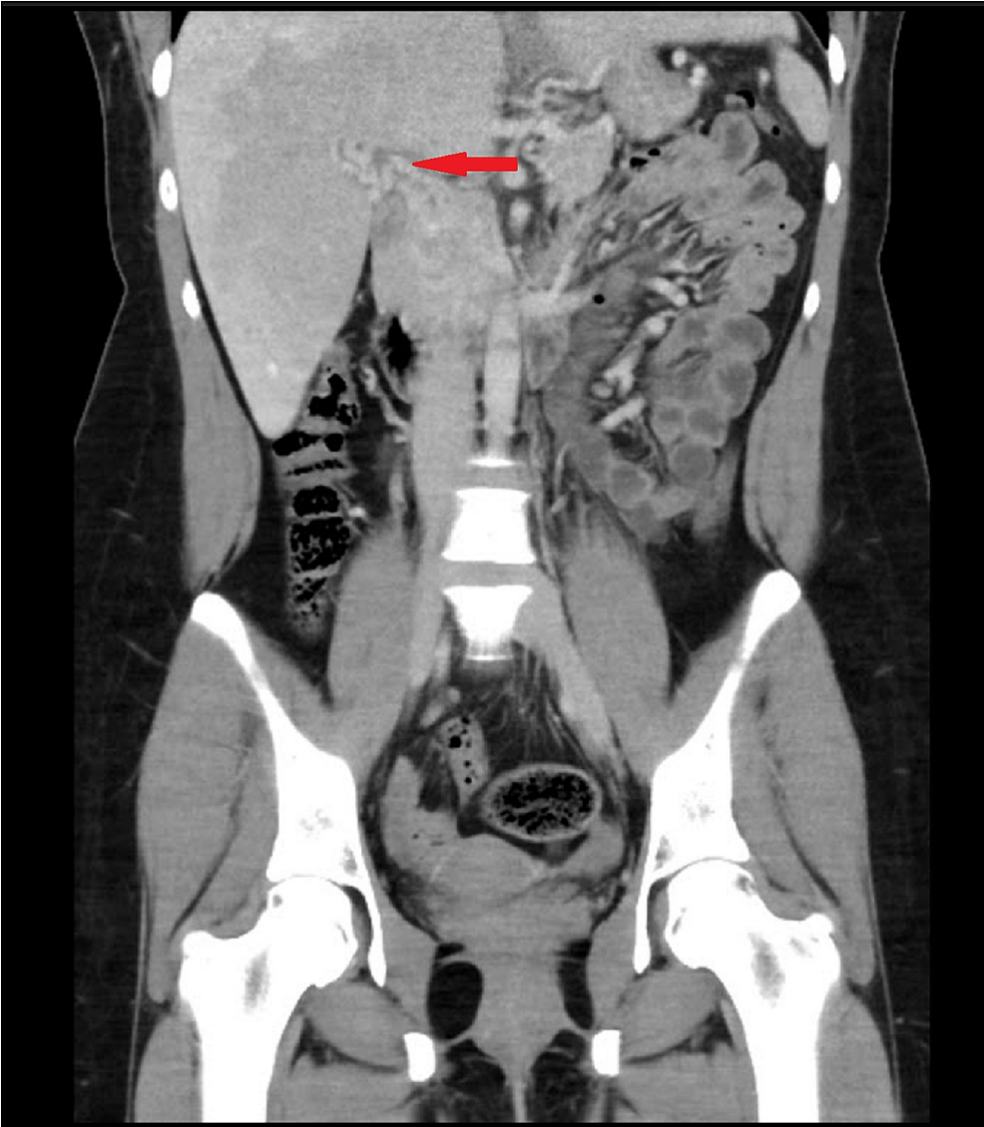
Progress CT scan CT Imaging six months after diagnosis showing persistent PVT with cavernous transformation of portal vein

A progress CT abdomen (Figure 2) was performed six months after initiation of anticoagulation. This scan showed cavernous transformation of the portal vein with small tubular enhancement. The splenic hilar varices were persistent. The gallbladder and the liver remained normal.

A gastroscopy was performed to look for oesophageal or gastric varices as well as screening for any malignancies that could account for the thrombosis. This was completed and no abnormalities were found.

The patient was discharged from the vascular surgeons with GP follow-up only as no surgical intervention was required. The haematologist also did not require any further investigations and follow-up with only the advice to remain on rivaroxaban indefinitely as this is the second episode of an idiopathic thrombotic event.

## Discussion

Thrombosis is the process of clot formation inside vessels. There are three main groups of risk factors for clot formation and is classically described as Virchow’s triad. These three factors are hypercoagulability, blood stasis, and cellular injury [3]. Hypercoagulability refers to the presence of a coagulation disorder such as Factor V Leiden. Typically, hypercoagulability screen involves testing for antithrombin III, protein C and S, lupus anticoagulant, Factor V Leiden, prothrombin gene mutation homocysteine, and coagulation parameters such as prothrombin time, international normalized ratio, activated thromboplastin time, fibrinogen, and platelets. Blood stasis refers to the disturbance of normal flow within a vessel. The cellular or endothelial injury occurs when there is trauma to vessels including those that are intentional such as in operations and unintentional injuries. Patients who develop thrombi usually have disturbances in one or more of the above domains. Therefore, investigation and management of thrombotic events are directed not only at the reduction of thrombi progression but also to identifying possible underlying causes to prevent future episodes.

PVT is a condition involving thrombotic occlusion of the portal vein. The occlusion can be partial or complete. Presentations of PVT can vary ranging from asymptomatic cases that are found incidentally on imaging or symptomatic cases with abdominal pain and fevers being the most common symptoms. Portal hypertension is also a common complication seen in chronic PVT but whilst portal hypertension itself does not manifest clinically, patients commonly present with its complications. Most commonly patients have splenomegaly, ascites and can present acutely with haemorrhaging from either oesophageal or rectal varices [4]. 

However, there are less common causes of venous thrombosis that the haematologist investigated for in this patient. Paroxysmal nocturnal haemoglobinuria (PNH) can lead to thromboembolic complications at a rate of 31% [5] and particularly affects the intra-abdominal veins. The hepatic and portal veins have been known to be affected with thrombosis accounting for up to 40.7% and 10.2% of thrombotic events in PNH respectively [6]. Although the PVT in this patient is unlikely to be from PNH, thrombotic events may carry high mortality rates if undiagnosed, with estimated mortality rates of 40-67% of PNH related deaths [7], hence the need to exclude this diagnosis. Another cause of thrombosis are JAK-2 mutations and although there have been some reported cases of this in the literature the exact incidence or prevalence is not known [8,9].

Malignancies must be considered in the face of PVT with unknown causes. At the time of diagnosis, the patient’s cervical screening was up to date with no prior abnormalities. Breast cancer was considered and after consulting breast screening services, the patient was advised that no immediate screening was required and she can start screening at age 40. Gastroscopy was completed with no upper gastrointestinal tract abnormalities identified. Colonoscopy was not completed. Alpha-fetoprotein levels were normal and combined with CT imaging, hepatocellular malignancy was excluded. 

Infections such as the hepatitis virus, cytomegalovirus (CMV) or Epstein-Barr virus have been known to increase the risk of splanchnic vessel thrombosis and if no known cause of PVT is identified, testing of these microbes may be considered [10]. It has been reported that even in the immunocompetent population, infections such as CMV can cause PVT [11]. Due to the lack of infectious symptoms and up-to-date vaccination status for hepatitis, no infectious screen was done during the workup of this patient. 

Currently, there are multiple imaging modalities that can be employed to investigate portal hypertension and cirrhosis. For this patient, CT and USG imaging was performed to investigate her initial presenting symptoms as they were easily accessible at the institution. Previous studies had estimated sensitivity for USG imaging to be 78.7% to 82.2% and for CT, 77.1% in identifying cirrhosis [12]. No further imaging was warranted for this patient as there were no abnormalities noted on both the CT and USG apart from the thrombosis that required further investigations.

At present, there are no clear guidelines detailing the recommended management of PVT in the context of non-cirrhotic and non-malignant causes. However, some studies have demonstrated that anticoagulation or early anticoagulation reduces the risk of progression and complications. Choudhry et al. [13] reported that portal vein recanalization was higher and death rates lower in those who were anticoagulated, although noting that the studied population group had PVT secondary to inflammatory disease. Plessier et al. [14] and Hall et al. [15] showed that early anticoagulation increased recanalization and reduced bleeding risk.

Another aspect of this patient’s management is follow-up and monitoring for potential complications. Complications can be chronic in the case of portal hypertension or less commonly occur acutely in the case of intestinal infarction, which carries a high mortality rate of up to 60% [16]. More frequently, complications arise secondary to portal hypertension, most clinically significant are varices from portosystemic shunting. Other less common complications such as ascites, hypersplenism and portal vein cavernoma can also occur. There is also much variation in follow-up durations and timing in the literature with no established protocols for reassessment. Hall et al. noted that follow-up periods were variable with some studies reviewing patients between zero to six years, some at 12 months or more, and some at less than 12 months. The surveillance interval for a patient ultimately needs to be determined on a case-to-case basis as more severe complications may warrant more frequent follow-up compared to those who have a stable and asymptomatic thrombus.

## Conclusions

PVT is most commonly seen in patients with underlying hepatobiliary or abdominal pathologies. Therefore, its diagnosis warrants further workup to exclude underlying conditions. Furthermore, specialist advice from a vascular surgeon and a haematologist should be sought regarding the management and investigation of the thrombosis. Close coordination of care from the patient's primary health carer is also vital in order to organize age and risk-appropriate malignancy screening as well as ongoing monitoring of any complications that may develop from unresolved PVT. In rare circumstances, no obvious causes can be identified as the trigger for the thrombotic event. From the case presented, it is evident that the literature around idiopathic PVTs are scarce and thus this condition warrants further research so that guidelines for anticoagulation and follow-up can be established. 
